# Long-term functional outcome in adult prison inmates with ADHD receiving OROS-methylphenidate

**DOI:** 10.1007/s00406-012-0317-8

**Published:** 2012-04-21

**Authors:** Ylva Ginsberg, Tatja Hirvikoski, Martin Grann, Nils Lindefors

**Affiliations:** 1 Department of Clinical Neuroscience, Division of Psychiatry, Karolinska Institutet, M59, 141 86 Stockholm, Sweden; 2 Department of Molecular Medicine and Surgery, Center for Molecular Medicine, Karolinska Institutet, Stockholm, Sweden; 3 Department of Medical Epidemiology and Biostatistics, Centre for Violence Prevention, Karolinska Institutet, Stockholm, Sweden; 4 Swedish Prison and Probation Service, Head Office, Norrköping, Sweden

**Keywords:** Adult, Attention deficit hyperactivity disorder, Long-term treatment, Methylphenidate, Prison inmates, Self-rating, Investigator-rating, Cognition, Executive functioning, Working memory, Motor activity, Actigraphy, Quality of life

## Abstract

In a recent randomized, double-blind, placebo-controlled trial, we established a robust efficacy (Cohen’s *d* = 2.17) of osmotic release oral system-methylphenidate (OROS-methylphenidate) delivered 72 mg daily for 5 weeks versus placebo on attention deficit hyperactivity disorder (ADHD) symptoms, global severity and global functioning in 30 adult male prison inmates with ADHD and coexisting disorders. Outcomes continued to improve during the subsequent 47-week open-label extension with OROS-methylphenidate delivered at a flexible daily dosage of up to 1.3 mg/kg body weight. In the present study, we evaluated long-term effectiveness and maintenance of improvement over the cumulated 52-week trial on cognition, motor activity, institutional behaviour and quality of life. Post hoc, we explored the associations between investigators’ and self-ratings of ADHD symptoms and between ratings of symptoms and functioning, respectively. Outcomes, calculated by repeated measures ANOVA, improved from baseline until week 16, with maintenance or further improvement until week 52. Both verbal and visuospatial working memory, and abstract verbal reasoning improved significantly over time, as well as several cognition-related measures and motor activity. No substance abuse was detected and a majority of participants took part in psychosocial treatment programmes. The quality of life domains of Learning, and Goals and values improved over time; the latter domain was at open-label endpoint significantly related to improvements in attention. Investigators’ and self-ratings of ADHD symptoms, as well as global symptom severity related most significantly to global functioning at week 52. Finally, investigators’ and self-ratings of ADHD symptoms associated significantly at baseline with increasing convergence over time.

## Introduction

Attention deficit hyperactivity disorder (ADHD) is a common, inherited disorder, which arises during childhood and frequently persists into adulthood. ADHD is characterized by developmentally inappropriate levels of inattention, hyperactivity and impulsivity [1]. Behavioural symptoms and cognitive deficits associated with ADHD have been extensively explored, especially in children. However, it is less known to what extent treatment ameliorates these associated cognitive deficits [2]. Traditionally, symptoms of hyperactivity have been assumed to decline by age and to change from gross motor overactivity as commonly observed in children, to a more subtle sense of inner restlessness in adults with ADHD [3]. However, increased levels of motor activity have recently been observed in adults with ADHD by means of objective measurements, contradicting the view of motor activity not being of a concern in adults [4]. There has also been a shift in the understanding of cognitive deficits associated with ADHD, from earlier theories suggesting a core deficit in response inhibition as part of a frontal lobe dysfunction, to an explanation of multiple cognitive deficits [2]. This new understanding is supported by the observed heterogeneity of cognitive impairments seen in ADHD samples, and the observation of executive function deficits, such as impairments in working memory, organizing and planning—although common, not being present in all individuals with ADHD [5, 6]. Despite the evidence of a strong genetic contribution to ADHD, results of candidate gene associations have yielded inconsistent results, suggested to be reflective of the heterogeneity and complexity of ADHD. Therefore, it has been proposed that the use of neuropsychological endophenotypes might facilitate in the detection of genetic effects [7]. Further, neuroimaging studies have demonstrated structural differences and deficits in the activation of several brain areas, and studies using neurophysiology have suggested a disturbed neuronal inhibition within individuals with ADHD [2, 8–11]. The functional impairments of ADHD related to core symptoms and cognitive deficits, affect several aspects of daily functioning, such as education, work performances, social relationships and quality of life [1, 12, 13]. Also, almost 80 % of adults with ADHD present with coexisting psychiatric disorders [5, 14]. Among these, substance use disorder and antisocial personality disorder are common, both increasing the risk of subsequent delinquency. Several studies estimate ADHD to be present among 25–45 % of adult prison inmates, as compared to about 2–5 % of adults in the general population [1, 15–17]. In prison inmates, ADHD is most often combined with externalizing symptoms of conduct disorder (CD), usually childhood-onset CD, but also adolescent-onset CD is reported. Recently, it has become evident that CD is the dominating risk factor in mediating later development of antisocial and delinquent behaviour, not ADHD alone [18]. It is estimated that about half of children with ADHD develop CD, and about half of them with CD subsequently develop antisocial personality disorder. In contrast to DSM-IV, the classification system of ICD-10 defines a category of hyperkinetic disorder of social behaviour (F90.1), thus differentiating between ADHD and ADHD with CD [18].

Treatment with methylphenidate demonstrates short-term efficacy in improving core symptoms of ADHD and is therefore often considered the drug of first choice, both in children and in adults [5, 19, 20]. Reports of long-term effectiveness of stimulants in children show mixed results. The MTA study conducted in children with ADHD reported temporary long-term effects that dissipated over time [21], as opposed to studies reporting long-term benefits of treating youths with ADHD [22, 23]. Reports of long-term effectiveness of stimulants in adults with ADHD are even sparser. These long-term studies mostly comprise open-label extensions of controlled short-term trials, but also long-term controlled trials and observational studies of clinical cases are reported. Taken together, the limited data suggest maintenance of treatment response to stimulants over 6 months—2 years, without developing tolerance of treatment effects [24–29]. However, more data on long-term effects in different study populations are warranted to clarify and differentiate between long-term effects and effects of long-term treatment.

In addition, participants of clinical pharmacological trials typically demonstrate less functional impairments, lower rates of lifetime coexisting psychiatric disorders, and higher occupation and socioeconomic status than individuals with ADHD seen in clinical practice. These notions suggest that results from many clinical trials may have limited external validity [30].

Moreover, the few studies that have evaluated methylphenidate treatment in participants with ADHD and coexisting substance use disorder could not establish efficacy as compared with placebo in improving ADHD symptoms [31]. And despite the high prevalence of ADHD in prison inmates, pharmacological treatment has not previously been evaluated in this group. Further, most trials have primarily evaluated effectiveness of pharmacotherapy on ADHD core symptoms, global functioning and global severity in the short term, with limited information regarding effects on cognition, and long-term outcomes of stimulant treatment in individuals with ADHD [2]. Most evaluations so far have been conducted in children, with reports of larger improvements on tasks without an executive component than on those with executive components [32, 33]. According to these studies, the optimal dose appears to vary across individuals, suggesting requirement of lower doses for improvement in cognitive symptoms than for behavioural ones. There has however not been any clear evidence of methylphenidate fully correcting cognitive deficits related to ADHD [34]. Studies evaluating effects of stimulants on neuropsychological performances in adult ADHD have shown mixed results [2, 35]. Briefly, the most consistent finding is improvement in vigilance or sustained attention [2, 35]. So far, only a few studies have evaluated the association between symptomatic improvements and improvements in daily functioning by ADHD treatment. These studies have suggested a translation of symptomatic improvements into functional improvements [27, 36–38]. This translation might be understood in such a way that individuals that become more attentive, structured and patient as a result of treatment, improve their ability to interact with family members, friends and co-workers, thus increasing their levels of social and daily functioning. Further, based on the observation of a correlation between investigators’ and self-ratings of ADHD symptoms in adults [39], it could be suggested that self-ratings would be reliable enough to replace investigators’ ratings. However, this question need to be further explored. We recently reported a randomized, double-blind, placebo-controlled 5-week trial, followed by 47-week open-label extension, conducted in 30 adult male prison inmates with ADHD and coexisting disorders [40]. When designing this trial, we aimed at increasing the external validity of results by allowing for participants with ADHD and coexisting disorders, thus being representative for a prison population.

Further, as this was, to our best knowledge, the first controlled trial of stimulants for ADHD, conducted within a prison setting, we aimed at gathering a broader range of information regarding treatment effects. Therefore, we assessed outcomes of symptoms, functioning, cognition, institutional behaviour, quality of life, adverse events and vital signs, both in the short-term and in the long-term. OROS-methylphenidate delivered 72 mg daily significantly improved ADHD core symptoms, global severity and global functioning versus placebo. All 30 participants entered the subsequent 47-week open-label extension without comparator. During this phase, OROS-methylphenidate delivered at a flexible daily dosage of up to 1.3 mg/kg body weight further improved outcomes within participants [40].

The present paper reports secondary outcomes of this cumulated 52-week trial. We evaluated the long-term effectiveness and maintenance of treatment effects from OROS-methylphenidate on cognition, motor activity, institutional behaviour and quality of life, from baseline until end of treatment after 52 weeks. Post hoc, we explored the relationships between ratings of symptoms and daily functioning, and between investigators’ and self-ratings of ADHD symptoms, respectively. We hypothesized that OROS-methylphenidate would improve aspects of cognition, motor activity, institutional behaviour and quality of life and that improvements would maintain over the entire 52-week study period. Finally, we hypothesized that ratings of symptoms and functional outcomes, as well as investigators’ and self-ratings of ADHD symptoms would be significantly associated.

## Methods

### Participants

Adult male prison inmates confirmed with ADHD took part in the present study. All participants were hosted at Norrtälje Prison, located outside Stockholm, Sweden. This high-security prison hosts primarily long-term, adult male inmates convicted of drug-related or violent crimes. The initial screening survey and diagnostic assessments were previously reported in detail [15]. All assessments were performed by experienced board-certified psychiatrists and clinical psychologists. Briefly, ADHD was confirmed by a clinical interview assessing symptoms and impairments of ADHD during both childhood and adulthood, in consistent with DSM-IV criteria [41]. Diagnostic assessments also included collection of information from parents, school records, health services, and the prison and probation service, regarding developmental history, current symptoms and impairments. Coexisting disorders were evaluated by the Structured Clinical Interview for DSM-IV Axis I Disorders (SCID I) [42], the Hare Psychopathy Check List-Revised (PCL-R) [43] and the SCID II Patient Questionnaire (SCID II PQ), a self-rated version of the Structured Clinical Interview for DSM-IV Axis II Personality Disorders [42]. Additional assessments included obtainment of medical history, physical examination, routine laboratory tests, supervised urine drug screening and neuropsychological tests assessing IQ and executive functions. When appropriate, assessments were extended for confirming autism-spectrum disorder in consistence with DSM-IV criteria [41].

Participants randomized to the clinical trial had to be established with ADHD in consistence with DSM-IV and to agree not to behave violently during the trial. Coexisting disorders, such as anxiety, depression and autism-spectrum disorder, were allowed. Previous drug-elicited episodes of psychosis or psychopathy as defined by Hare (total sum-score ≥30) were not a cause for exclusion. Concurrent medication not interfering with methylphenidate was allowed for treating coexisting disorders, as long as doses were kept stable for at least 4 weeks at baseline. Pharmacological treatment interfering with methylphenidate had to be tapered off in advance to the baseline visit. Also, participants had to be confirmed without substance abuse during the preceding 3 months and should not fulfil the diagnostic criteria for mental retardation or for any serious medical illness. However, participants with hepatitis C without liver insufficiency could take part in the trial. Full details of the study design, inclusion and exclusion criteria have been reported previously [40].

### Study design

This study (ClinicalTrials.gov:NCT00482313) was a randomized, double-blind, placebo-controlled parallel-group 5-week trial, followed by a 47-week open-label extension. It was conducted between May 2007 and April 2010 in 30 adult male prison inmates. Participants were randomly assigned to placebo or OROS-methylphenidate at a ratio of 1:1. The study was approved by the Ethical Board of Stockholm, Sweden (2006/1141-31/3), and by the Swedish Medical Products Agency (EudraCT-nr 2006-002553-80), respectively. All participants provided written informed consent after they had received a thorough description of the study. The trial was independently monitored by the Karolinska Trial Alliance and inspected by the Swedish Medical Products Agency to validate adherence to Good Clinical Practice and the Declaration of Helsinki.

### Study intervention

The study drug was titrated from 36 mg daily for 4 days to 54 mg daily for 3 days and then to 72 mg daily for the remaining 4 weeks. All participants that completed the 5-week trial were eligible to enter the 47-week open-label extension, starting the day after completion of the 5-week phase. During the open-label extension, OROS-methylphenidate was individually titrated from 36 mg daily to an optimal dose, on the basis of response and tolerability, with a maximum daily dose of 1.3 mg/kg body weight. In case of intolerable adverse events, lower doses were administered, followed by increased doses once participants recovered from the adverse event. In addition to study medication, participants were, as part of regular prison routines, provided educational activities and accredited treatment programmes. However, these psychosocial interventions did not specifically address symptoms and associated impairments of ADHD.

### Assessments

Assessments of self-reported Quality of Life Inventory (QOLI) [44], as well as neuropsychological assessments performed by certified psychologists, were conducted at baseline (T1), at study week 16 (T2) and at endpoint study week 52 (T3), with the exception of the WAIS-III subtest Similarities [45], which was assessed at baseline (T1), and at study week 52 (T3) only. Information regarding participation in educational activities was recorded by the teachers, whereas correctional officers recorded what treatment programmes participants took part in, as well as critical incidents that occurred for each participant throughout the study.

### Outcome measures

#### The Digit Span and the Span Board

Changes in verbal working memory capacity were measured by the Digit Span, a subtest of WAIS [45]. The corresponding non-verbal task Span Board measured changes in visuospatial working memory [46]. Results of both tests are expressed on age-scaled scores, with a population mean of 10 (*M* = 10) and a standard deviation of 3 (SD = 3). For further analyses of different aspects of working memory performances, we divided the results of both tests into forward and backwards performances, respectively. Forward performances are associated with *maintenance* of information in working memory. On the other hand, backwards performances relate to both *maintenance* and *manipulation* of information in working memory, thus comprising a more demanding task. These divided measures were reported as number of correctly indicated series.

#### Similarities

The WAIS-III subtest Similarities [45] is a measure of abstract verbal reasoning. Similarities is not expected to show learning effects from repeated testing, especially not with long test–retest interval, as in the present study. It was used as a specificity measure for the assessments performed during the study, thus only administered at baseline (T1) and at study week 52 (T3).

#### The Conners’ Continuous Performance Test II

The Conners’ Continuous Performance Test II (Conners’ CPT II) [47] is a computerized visual continuous performance test (CPT). During this 14-min lasting go/no go test, letters are presented on a computer screen and the participant is instructed to respond both accurately and fast by pressing a button for each letter except the letter “X”. Measures are grouped into those reflecting functions such as *basic reaction time*, *variability* and *accuracy.* Results of the CPT are expressed as T-scores, corresponding to a population norm with *M* = 50 and SD = 10. Shortly, higher scores reflect poorer performances.

#### The QbTest

The QbTest combines a simultaneous delivered computerized visual CPT with a high-precision infrared motion tracking device (provided by Qbtech, Stockholm, Sweden; www.qbtech.se/products/qbtest; QbTest technical manual, Fredrik Ulberstad, Rev E, January 2012). Motions are captured and recorded by a reflective headband marker, with a sampling rate of 50 times per second and a spatial resolution of 1/27 mm per infrared camera unit. The test duration is 20 min, but to adjust for test adaptation, only data from the last 15 min are analysed. Four different types of stimuli, varying in colour (blue, red) and shape (square, circle) are presented on the computer screen in a pseudorandom order. The participant is instructed to react as fast and accurate as possible and press a button when the currently presented stimulus matches with the stimulus presented directly before, in both shape and colour. Otherwise, the participant is instructed not to press the button, corresponding to inhibiting the motor response. This 1-back working memory task used in the adult version of QbTest is more challenging than the go/no go task provided by the Conners’ CPT II and therefore suggested to be a more appropriate task for adults. The QbTest measures are grouped into those related to *motor activity* and *cognition*, as presented in Table 4. Due to skewed distribution of data, raw scores are transformed to age- and sex-scaled Q-scores, corresponding to z-scores (norm population *M* = 0, SD = 1). QbTest demonstrates good test–retest reliability (Ulberstad F, 2011, data on file). Shortly, higher scores reflect poorer performances.

#### Institutional behaviour

Institutional behaviour was evaluated in several ways. As part of regular prison routines, inmates are obliged to participate in scheduled programmes during daytime (www.kriminalvarden.se). These programmes comprise activities such as vocational training, educational programmes and participation in evidence-based treatment programmes, aiming to increase the chances in obtaining a job, as well as preventing from continued substance abuse or return to crime after served conviction. All treatment programmes are accredited by the Swedish Prison and Probation Service. Each participant was provided an individualized combination of programmes decided by the participant’s assessed risks and needs. During the present study, general offending programmes (OTO—One To One, ETS—Enhanced Thinking Skills), programme for violence prevention (aggression replacement training), substance abuse programmes (dare to choose, PRISM—Program for reducing individual substance misuse, twelve-step programme), sexual offending programme (ROS—Relations and companionship) and motivational programme (behaviour–talk–change) were provided. Educational programmes adhering to the Swedish curriculum were provided by teachers at the Learning center of Norrtälje Prison, with the purpose of increasing basic skills such as reading, writing and mathematics. Educational studies were preferably provided at the primary school level, but it was also possible to study at high school level or to continue university studies when appropriate. Information regarding participation in treatment programmes and educational activities was collected by correctional officers and teachers, respectively. Results were reported by descriptive statistics.

Diversion of drugs is a matter of concern within many prison settings. To control for substance abuse within participants during the course of the study, supervised urine drug screening was regularly performed by correctional officers at the prison wing. Results of the drug screening procedures were reported by descriptive statistics. Finally, critical incidents that occurred during the course of the study were recorded by prison officers and compared with the number of incidents recorded during the corresponding time period preceding the randomization of the participant.

#### The Quality of Life Inventory

Self-rated quality of life was assessed by a cross-cultural validated Swedish version of the Quality of Life Inventory (QOLI) [44]. This general, 32-item self-administered rating scale is considered applicable to both non-psychiatric and psychiatric populations. It measures satisfaction and importance of 16 different domains, reflecting areas of achievement, social functioning, personal growth and surroundings [48, 49]. QOLI is shown to be reliable, valid and sensitive to treatment-related changes in several clinical populations [49, 50]. However, to our best knowledge, quality of life measured by QOLI has not previously been reported for ADHD populations.

The participant rates the degree of importance of each life area to their overall happiness and satisfaction from 0 = *not at all important* to 2 = *very important*, and its satisfaction with each domain from −3 = *very dissatisfied* to +3 = *very satisfied*, excluding 0. The importance and satisfaction scores of each life area are then multiplied to create 16 weighted satisfaction scores. A global index of subjective quality of life, expressed as a total T-score, comprises the sum of the weighted satisfaction scores in all areas rated as important by the participant. However, since life satisfaction may differ between specific domains, and several domains were considered non-relevant or difficult to influence within a restricted prison environment, we decided to evaluate changes in the different domains instead of using the single global index of life satisfaction.

#### The Conners’ Adult ADHD Rating Scale-Observer: Screening Version

The investigator-rated Conners’ Adult ADHD Rating Scale-Observer: Screening Version (CAARS: O-SV) [51] comprises 18 items corresponding to the 18 DSM-IV ADHD symptom criteria. This scale provides a total sum-score, based on ratings of symptom frequencies from *0* = *not at all*, to *3* = *very much/very frequently* (range 0–54). The 18 items can be further divided into a 9-item subscale of inattention (range 0–27), and a 9-item subscale of hyperactivity/impulsivity (range 0–27), respectively.

#### The Adult ADHD Self-Report Scale

In the Adult ADHD Self-Report Scale (ASRS) [51], 18 items corresponding to the 18 ADHD symptom criteria of DSM-IV are worded to be more reflective of the expression of ADHD symptoms in adulthood. The participant rates the frequency of each symptom from *0* = *never* to *4* = *very often*, providing a total sum-score (range, 0–72). ASRS can be further divided into a 9-item subscale of inattention (range, 0–36) and a 9-item subscale of hyperactivity/impulsivity (range, 0–36), respectively.

#### The Clinical Global Impression Severity of Illness Scale

The investigator used the Clinical Global Impression-Severity Scale (CGI-S) [52] to rate the participant’s global symptom severity of ADHD on a 7-point scale, ranging from *1* = *not ill* to *7* = *extremely severe.*

#### The Global Assessment of Functioning Scale

The investigator used the Global Assessment of Functioning Scale (GAF) [53] to rate the participant’s global functioning on a visual analogue scale, ranging 0–100. A higher value reflects an increased level of functioning as compared to a lower value.

### Statistical analysis

The primary outcome measure of the clinical trial was the change in investigator-rated ADHD symptoms from baseline until end of week 5 in the double-blind phase, measured by the total sum-score of CAARS: O-SV. The sample size was based on this primary outcome measure. Details of the sample size calculation, as well as results of CAARS: O-SV and secondary outcomes of global severity and global functioning, were reported previously, together with details of adverse events and vital signs during the cumulated 52-week trial [40].

In this secondary analysis, we employed repeated measures analysis of variance (rmANOVA) for treatment effects within participants on cognition-related measures and measures of quality of life. Analyses were presented in two ways, including (1) participants with complete data from all assessments (per-protocol population) and (2) the intent-to-treat population (ITT), defined as all randomized participants providing baseline data. Last observation carried forward (LOCF) was used for imputation of missing data. Single missing values were handled conservatively by substituting the missing value with the higher value from the preceding or following visit. The effect size was presented by partial eta squared (η_p_^2^) for efficacy measures and interpreted using the guidelines as proposed by Cohen; 0.01 = small effect size, 0.06 = moderate effect size, and 0.14 = large effect size [54]. We expected the largest changes in cognition and motor activity to occur between baseline (T1) and study week 16 (T2). However, we also evaluated changes between study week 16 (T2) and study week 52 (T3), by performing tests of within-subjects contrasts, with simple contrasts using T3 as the reference level. Significance levels and confidence intervals were adjusted with Bonferroni corrections in the analyses of changes in QOLI domains (0.05/16). Institutional behaviour was reported by descriptive statistics. Alpha level was set at *P* = 0.05 (two-sided significance).

Further, post hoc analyses were performed to explore the relationships between investigator-rated (CAARS: O-SV) and self-rated (ASRS) improvements in ADHD symptoms and between symptomatic (CAARS: O-SV subscales, ASRS subscales, CGI-S) and functional (GAF, QOLI domains) ratings, based on completers. Due to the small sample size, exploration of relationships between rating scales was limited to the determination of bivariate correlation coefficients. After checking for normality, Pearson’s product moment correlation coefficients (*r*) were calculated for scores, as well as for changes in scores of rating scales at baseline, study week 16 and open-label endpoint at week 52.

## Results

### Participants

Baseline data demonstrated that coexisting disorders were common; lifetime substance use disorder was reported by all participants, all but one confirmed antisocial personality disorder, a majority were established with mood and/or anxiety disorders, and one-quarter confirmed concomitant autism-spectrum disorder. At study entry, almost half of participants received pharmacological treatment for mood and/or anxiety disorders. Scores of rating scales revealed that participants were substantially symptomatic and impaired from ADHD at baseline (Table 1).

**Table 1:** Baseline demographics, clinical characteristics and baseline scores for randomized participants

	Randomized participants (*n* = 30)
Age (years)	
Mean, SD	34.4 (10.67)
Range	21–61
Gender, male, *n* (%)	30 (100)
Educational level, 9 year compulsory school, or less, *n* (%)	25 (83)
Educational support during childhood, *n* (%)	24 (80)
Full scale IQ, (*N* = 22)	
Mean, SD	95.18 (9.99)
Range	78–113
Adult ADHD subtype^a^, *n* (%)	
Combined type	28 (93)
Predominantly inattentive	2 (7)
Autism-spectrum disorder^a,b^, *n* (%)	7 (23)
Mood- and/or anxiety disorder^a^, lifetime, *n* (%)	22 (73)
Conduct disorder^a^	30 (100)
Personality disorders^a,c^, (*N* = 23)	
Antisocial, *n* (%)	22 (96)
Borderline, *n* (%)	17 (74)
Paranoid, *n* (%)	17 (74)
Narcissistic, *n* (%)	15 (65)
Obsessive-compulsive, *n* (%)	12 (52)
Passive-aggressive, *n* (%)	11 (48)
Avoidant, *n* (%)	11(48)
Depressive, *n* (%)	8 (35)
Dependent, *n* (%)	7 (30)
Schizotypal, *n* (%)	5 (22)
Schizoid, *n* (%)	2 (9)
Histrionic, *n* (%)	0 (0)
Substance use disorder^a^, lifetime, *n* (%)	30 (100)
Preferred drug of choice, *n* (%)	
Alcohol	4 (13)
Amphetamine	18 (60)
Cocaine	4 (13)
Cannabis	1 (7)
Opioids	1 (7)
Anabolic steroids	1 (7)
Other	1 (7)
Psychopathy^d^	3 (10)
Treatment for psychiatric disorders at baseline visit, *n* (%)	13 (43)
CAARS: O-SV^e^, baseline sum-score, mean, 95 % CI	40.0 (38.1–41.8)
ASRS^f^, baseline sum-score, mean, 95 % CI	55.3 (52.0–58.6)
GAF^g^, baseline total score; mean, 95 % CI	35.2 (33.3–37.1)
CGI-Severity^h^, baseline score, mean, 95 % CI	5.9 (5.7–6.1)
Marked, *n* (%)	6 (20)
Severe, *n* (%)	21 (70)
Extremely severe, *n* (%)	3 (10)

^a^Diagnosis in accordance to DSM-IV

^b^Autism-spectrum disorders includes Asperger syndrome and pervasive developmental disorders, not otherwise specified (PDD-NOS)

^c^Frequencies of personality disorders were estimated by increasing the cut-off level for each personality disorder by one score on the SCID II Personality Questionnaire to equal the cut-off score of the SCID II Interview

^d^Psychopathy was defined as a total sum-score of ≥30 by the Psychopathy Check List-Revised (PCL-R)

^e^Conners’ Adult ADHD Rating Scale-Observer: Screening Version

^f^Adult ADHD Self-Report Scale

^g^Global Assessment of Functioning Scale

^h^Clinical Global Impression-Severity Scale

All 30 randomized male prison inmates, aged 21–61, completed the initial 5-week randomized, double-blind, placebo-controlled trial and entered the 47-week open-label extension.

**Fig. 1:**
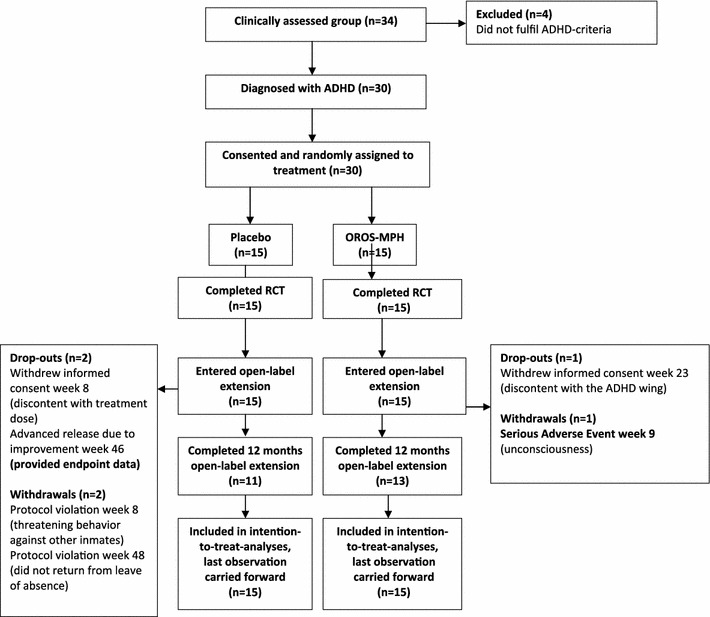
Study flow chart of participants in the cumulative 52-week trial

A total of 24 participants completed the cumulated 52-week trial, as seen from the study flow chart presented in Fig. 1. However, 25 participants provided endpoint data, as one participant was transferred from Norrtälje Prison in advance due to improvement and therefore underwent endpoint assessments at study week 46. Full details of the initial 5-week trial and some of the secondary analyses from the open-label extension were previously reported [40].

Briefly, during the initial double-blind phase, OROS-methylphenidate significantly improved ADHD symptoms (CAARS: O-SV, *P* < 0.001; Cohen’s *d* = 2.17; ASRS, *P* = 0.003), global symptom severity (CGI-S, *P* < 0.0005) and global functioning (GAF, *P* < 0.0005). Numbers needed to treat (NNT) was 1.1 (95 % CI, 1–2), and the placebo response was observed to be non-significant. ADHD symptoms, global severity and global functioning continued to improve during the open-label extension phase without comparator. At study endpoint at week 52, the mean dose of OROS-methylphenidate was 105 (SD = 27.2) mg daily or 1.22 (SD = 0.28) mg/kg body weight daily, based on the ITT-population. One serious adverse event of unknown cause occurred during the open-label extension, which justified study withdrawal. Apart from this event, treatment was generally well tolerated. Mucosal dryness was the only adverse event that occurred more frequently in the OROS-methylphenidate group than in the placebo group. Overall, the most frequently reported adverse events, considered as associated with OROS-methylphenidate, were abdominal discomfort, headache, mucosal dryness, depressed mood, loss of appetite, anxiety, diarrhoea, sweating, interrupted sleep and fatigue. The severity of adverse events was usually rated as mild to moderate and did not lead to discontinuation. There were no significant changes in blood pressure, heart rate or body weight during the initial placebo-controlled phase in either group. When considering the cumulated 52-week trial, the group that received OROS-methylphenidate from baseline, significantly increased both the systolic (21.5 mmHg; 95 % CI 8.9–34.0) and the diastolic (11.0 mmHg; 95 % CI 4.9–17.1) blood pressure, but there were no significant changes in the heart rate or body weight. On the other hand, in the group that received placebo during the initial phase, the heart rate increased significantly (13.2 beats per minute; 95 % CI, 7.0–19.4) over the cumulated 52-week period, whereas body weight, systolic and diastolic blood pressure remained almost unchanged.

### Outcome measures

#### The Digit Span and the Span Board

**Fig. 2:**
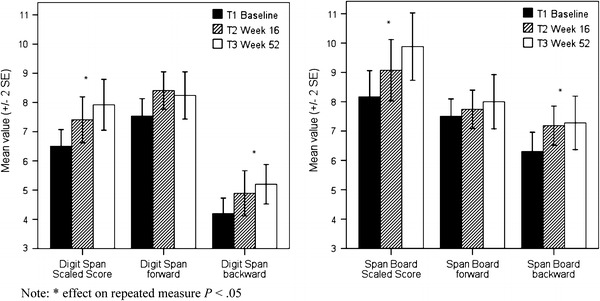
Both verbal working memory (Digit Span, *panel* to the *left*) and visuospatial working memory (Span Board, *panel* to the *right*) improved over time. The largest improvements were observed from baseline until study week 16. Data from completers (*n* = 25) are presented in the figure. Note: *Asterisk* indicates the effect on repeated measure *P* < 0.05

A total of 25 participants completed all assessments. Both verbal working memory measured by the Digit Span and visuospatial working memory measured by the Span Board improved significantly with large effect sizes within participants over time (Table 2; Fig. 2). Further, when analysing components of working memory, neither Digit Span forward nor Span Board forward improved significantly over time. In contrast, both backwards tasks considered to be more working memory demanding than the forwards tasks, improved across time. Digit Span backwards improved significantly, whereas Span Board backwards also improved but not significantly in completers (*P* = 0.06). However, when the ITT-population was analysed, Span Board backwards also improved significantly (Table 2; Fig. 2). Post hoc analyses demonstrated that working memory improved mainly between baseline and week 16 (T2), without any further significant improvements observed between week 16 (T2) and endpoint at week 52 (T3), as presented in Table 2.

**Table 2:** Statistics from repeated measures ANOVAs for completers in both working memory tests, Digit Span and Span Board

*n* = 25 (*n* = 30 ITT/LOCF)	*F* (*F* ITT)	*P* (*P* ITT)	η_p_^2^ (η_p_^2^ ITT)	Within-subject contrasts	
				*P* T1 vs. T3	*P* T2 vs. T3
Digit Span Scaled Scores	6.33 *(7.00)*	**0.004** ***(0.002)***	0.21 *(0.19)*	**0.007**	0.198
Digits forward	1.91 *(2.71)*	0.167 *(0.086)*	0.07 *(0.09)*	0.318	0.424
Digits backwards	4.45 *(4.73)*	**0.017** ***(0.013)***	0.16 *(0.14)*	**0.005**	0.518
Span Board Scaled Scores	5.16 *(5.72)*	**0.009** ***(0.005)***	0.18 *(0.17)*	**0.004**	0.162
Span forward	0.39 *(0.64)*	0.680 *(0.529)*	0.02 *(0.02)*	0.584	0.461
Span backwards	3.03 *(3.24)*	0.057 ***(0.046)***	0.11 *(0.10)*	**0.021**	0.942

Statistics for the intent-to-treat (ITT) population using last observation carried forward (LOCF) are presented within parentheses

Bold values indicate statistically significant *P* value

Italic values indicate the results of ITT-population

Bold italic values indicate statistically significant *P* values of the ITT-population

#### Similarities

The ability of verbal abstract reasoning, as measured by Similarities, improved significantly within participants with large effect sizes [54], between baseline (T1) and open-label endpoint at week 52 (T3), in both completers (*n* = 25) (*F* = 9.97, *P* = 0.004, η_p_^2^ = 0.29) and the ITT-population (*F* = 9.39, *P* = 0.005, η_p_^2^ = 0.25).

#### The Conners’ Continuous Performance Test II

**Fig. 3:**
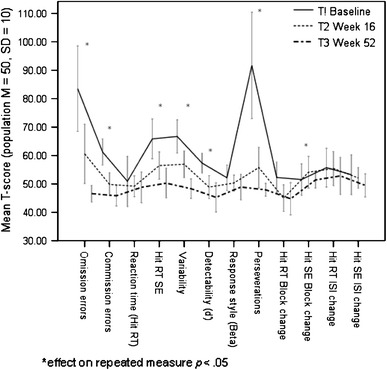
Seven out of twelve measures in the Conners’ Continuous Performance Test II improved. Five participants with extreme values at baseline (T-score >200) were excluded in the figure. Data from completers (*n* = 21) are presented in the figure; baseline data were missing for three participants due to technical error

A total of 21 participants completed all three assessments of Conners’ CPT II. As baseline data were missing for 3 participants due to technical error, the ITT-analyses using LOCF included 27 participants. The *reaction time* was normal within participants (*M* = 48.50, SD = 20.97) at baseline as compared to the norm, and no statistical changes were observed across the study period (Table 3; Fig. 3). Four out of 7 *variability-dependent* measures improved significantly over time, as presented in Table 3. Notably, as we observed extreme values (T-score > 200) for 5 out of 21 participants in Perseverations at baseline, they were considered as outliers. To avoid confounding effects, statistics were performed both with and without their values. However, Perseverations improved significantly also when excluding values of the outliers (*F* = 12.06, *P* = 0.002, η_p_^2^ = 0.43). A total of 3 out of 4 *accuracy-dependent* measures improved significantly over time (Table 3). In summary, OROS-methylphenidate improved 7 out of 12 Conners’ CPT II measures significantly, with large effect sizes as measured by η_p_^2^ [54]. The largest improvements were observed between baseline (T1) and study week 16 (T2), but some measures continued to improve significantly also between study week 16 (T2) and endpoint at study week 52 (T3), as depicted in Table 3.

**Table 3:** Statistics from repeated measures ANOVA of the Conners’ Continuous Performance Test II of completers, *n* = 21

*n* = 21 (*n* = 27 ITT/LOCF)	*F* (*F* ITT)	*P* (*P* ITT)	η_p_^2^ (η_p_^2^ ITT)	Within-subject contrasts	
				*P* T1 vs. T3	*P* T2 vs. T3
Hit reaction time	0.05 *(0.04)*	0.951 *(0.924)*	0.00 *(0.00)*	0.806	0.742
*Variability-dependent measures*					
Hit reaction time standard error	16.38 *(8.47)*	**<0.001** ***(0.003)***	0.45 *(0.25)*	**<0.001**	**0.014**
Variability	22.82 *(12.27)*	**<0.001** ***(*** **<** ***0.001)***	0.53 *(0.32)*	**<0.001**	**0.004**
Hit reaction time block change	7.99 *(5.17)*	**0.001** ***(0.013)***	0.29 *(0.17)*	**0.004**	0.914
Hit standard error block change	0.45 *(0.00)*	0.640 *(0.996)*	0.02 *(0.00)*	0.388	0.948
Perseverations	9.35 *(7.50)*	**0.006** ***(0.011)***	0.32 *(0.22)*	**0.005**	**0.048**
Hit reaction time inter-stimulus intervals change	0.43 *(0.36)*	0.651 *(0.695)*	0.02 *(0.01)*	0.529	0.386
Hit standard error inter-stimulus intervals change	0.50 *(0.064)*	0.539 *(0.854)*	0.02 *(0.00)*	0.450	0.518
*Accuracy-dependent measures*					
Omission errors	18.15 *(9.46)*	**<0.001** ***(0.002)***	0.48 *(0.27)*	**<0.001**	**0.032**
Commission errors	31.57 *(18.66)*	**<0.001** ***(*** **<** ***0.001)***	0.61 *(0.42)*	**<0.001**	0.071
Detectability	14.32 *(9.96)*	**<0.001** ***(*** **<** ***0.001)***	0.42 *(0.28)*	**<0.001**	0.255
Response style	1.40 *(1.03)*	0.257 *(0.341)*	0.07 *(0.04)*	0.234	0.675

Statistics from the intent-to-treat (ITT) population using last observation carried forward (LOCF) are presented within parentheses, *n* = 27; baseline data were missing for three participants due to technical error

Bold values indicate statistically significant *P* value

Italic values indicate the results of ITT-population

Bold italic values indicate statistically significant *P* values of the ITT-population

#### The QbTest

**Fig. 4:**
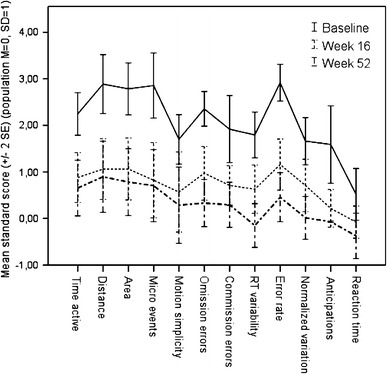
Motor activity and cognition-related measures improved significantly over time as measured by QbTest. Data from completers, *n* = 23 (one missing data due to technical error) and *n* = 24, respectively, are presented as age- and sex-scaled scores with a population mean of 0 (*M* = 0) and a standard deviation of 1 (SD = 1)

A total of 24 participants provided complete data on assessments of motor activity, and 23 participants (one missing data due to technical error) provided complete data on assessments of cognition by QbTest. Data are presented as raw scores in Table 4, and as age- and sex-scaled Q-scores in Fig. 4. Table 4 and Fig. 4 depict that all 5 measures related to *motor activity* improved significantly over time, as did all 7 *cognition*-*related* measures. Effect sizes expressed as η_p_^2^ were large for most measures [54], and the results of the per-protocol population and the ITT-population were almost the same, as evident from Table 4.

**Table 4:** Statistics from repeated measures ANOVA of the QbTest Continuous Performance Test of completers, *n* = 23 (one missing data due to technical error) and *n* = 24, respectively

	T1 M (±SD)	T2 M (±SD)	T3 M (±SD)	*F* (*F* ITT)	*P* (*P* ITT)	η_p_^2^ (η_p_^2^ ITT)	Within-subject contrasts	
							*P* T1 vs. T3	*P* T2 vs. T3
*Motor activity n* = *24 (n* = *30 ITT/LOCF)*								
Time active (%)	42.75 ± 26.83	15.13 ± 17.38	13.42 ± 15.13	22.23 *(16.98)*	**<0.001** ***(*** **<** ***0.001)***	0.49 *(0.37)*	**<0.001**	0.625
Distance (m)	23.33 ± 16.72	7.39 ± 5.73	8.50 ± 9.90	17.50 *(18.87)*	**<0.001** ***(*** **<** ***0.001)***	0.43 *(0.39)*	**<0.001**	0.515
Area (cm^2^)	86.08 ± 60.10	28.04 ± 28.56	26.88 ± 32.14	23.47 *(26.35)*	**<0.001** ***(*** **<** ***0.001)***	0.51 *(0.48)*	**<0.001**	0.807
Micro-events	10,892.83 ± 6,766.47	4,038.42 ± 3,249.16	3,996.96 ± 3,984.00	21.93 *(17.97)*	**<0.001** ***(*** **<** ***0.001)***	0.49 *(0.38)*	**<0.001**	0.958
Motion simplicity	48.68 ± 13.16	38.34 ± 15.74	35.75 ± 17.89	12.39 *(14.62)*	**<0.001** ***(*** **<** ***0.001)***	0.35 *(0.34)*	**<0.001**	0.261
*Cognition n* = *23 (n* = *30 ITT/LOCF)*								
Reaction time (ms)	598.39 ± 144.67	537.43 ± 83.71	510.83 ± 114.72	4.80 *(5.54)*	**0.024** ***(0.014)***	0.18 *(0.16)*	**0.028**	0.235
Reaction time variation (ms)	218.39 ± 63.58	166.70 ± 54.39	134.39 ± 47.33	23.03 *(25.17)*	**<0.001** ***(*** **<** ***0.001)***	0.51 *(0.47)*	**<0.001**	**0.005**
Normalized variation (%)	37.13 ± 9.16	30.87 ± 8.69	26.43 ± 7.20	17.50 *(20.98)*	**<0.001** ***(*** **<** ***0.001)***	0.44 *(0.42)*	**<0.001**	**0.007**
Omission errors (%)	37.75 ± 23.67	16.70 ± 21.78	8.54 ± 11.22	18.93 *(18.84)*	**<0.001** ***(*** **<** ***0.001)***	0.46 *(0.34)*	**<0.001**	**0.038**
Commission errors (%)	7.46 ± 10.48	1.42 ± 1.65	1.13 ± 1.50	8.36 *(7.53)*	**0.007** ***(0.009)***	0.28 *(0.21)*	**0.005**	0.483
Anticipatory errors (%)	2.04 ± 3.96	0.09 ± 0.29	0.00 ± 0.00	5.79 *(5.36)*	**0.025** ***(0.027)***	0.21 *(0.16)*	**0.022**	0.162
Error rate (%)	15.56 ± 10.16	5.30 ± 5.98	3.00 ± 3.15	26.28 *(24.93)*	**<0.001** ***(*** **<** ***0.001)***	0.56 *(0.46)*	**<0.001**	**0.027**

Statistics from the intent-to-treat (ITT) population using last observation carried forward (LOCF) are presented within parentheses, *n* = 30. Data in the table are presented as raw scores

Bold values indicate statistically significant *P* value

Italic values indicate the results of ITT-population

Bold italic values indicate statistically significant *P* values of the ITT-population

No further improvements were observed within participants between week 16 (T2) and week 52 (T3) in any measure related to *motor activity*. However, 4 out of 7 *cognition-related* measures improved further and significantly between study week 16 (T2) and open-label endpoint at week 52 (T3), as depicted in Table 4 and Fig. 4.

#### Institutional behaviour

A total of 24 participants out of 30 (80 %) took part in at least one accredited treatment programme. About two-thirds of them participated in two or three different programmes. The most frequently used programmes during this study were general offending programmes, provided individually and as a group intervention, as well as a violence preventing programme conducted in a group setting. A motivational programme and programmes preventing continued substance abuse were also common. All sexual offenders took part in a programme addressing relations and companionship. Also, a vast majority, 26 participants out of 30 (87 %) took part in educational programmes, mainly at the primary school level. Further, no side abuse (0 %) was detected during the study, as confirmed by supervised urine drug screening. Finally, we also explored the number of reported critical incidents during the 52 weeks preceding the trial. The number of reports was clearly reduced during the trial compared with the year before. However, most participants had spent the year before entering the trial in other prisons, and because we suspected there might be a methodological problem with different reporting practices, we did not pursue these analyses further with statistical tests.

#### Quality of Life Inventory (QOLI)

A total of 25 participants provided complete data on the self-rated QOLI, assessed at baseline (T1), week 16 (T2) and open-label endpoint at week 52 (T3). As presented in Table 5, quality of life improved significantly over time with a large effect size [54] in the specific domain of Goals and values, both in completers (*n* = 25) (*F* = 12.78, *P* < 0.001, η_p_^2^ = 0.53) and in the ITT-population (*n* = 30) (*F* = 10.41, *P* < 0.001, η_p_^2^ = 0.43). Quality of life also improved substantially in the Learning domain of both completers (*n* = 25) (*F* = 15.53, *P* < 0.001, η_p_^2^ = 0.58) and the ITT-population (*n* = 30) (*F* = 16.23, *P* < 0.001, η_p_^2^ = 0.54). As seen from Table 5, quality of life mainly improved between baseline and study week 16, with maintained improvements between study week 16 and week 52.

**Table 5:** Statistics from repeated measures ANOVA of the Quality of Life Inventory of completers, *n* = 25

	T1 M (SD)	T2 M (SD)	T3 M (SD)	*F* (*F* ITT)	*P* (*P* ITT)	η_p_^2^ (η_p_^2^ ITT)	Within-subject contrasts	
							*P* T1 vs. T3	*P* T2 vs. T3
*Weighted item, n* *=* *25 (n* *=* *30 ITT/LOCF)*								
1. Health	−0.70 (3.57)	0.93 (3.56)	1.16 (3.71)	4.12 (3.75)	0.030 (0.036)	0.26 (0.21)	0.022 (0.028)	1.000 (1.000)
2. Self-regard	−0.27 (3.46)	1.37 (3.35)	1.96 (2.91)	4.07 (3.79)	0.031 (0.035)	0.26 (0.21)	0.024 (0.028)	1.000 (1.000)
3. Goals and values	−0.97 (3.34)	1.41 (3.48)	2.36 (3.38)	12.78 (10.41)	**<0**.**001 (<0**.**001)**	0.53 (0.43)	**<0**.**001 (<0**.**001)**	0.613 (0.609)
4. Standard-of-living	−3.20 (2.98)	−2.11 (2.82)	−2.00 (2.75)	3.71 (1.50)	0.040 (0.240)	0.24 (0.10)	0.089 (0.445)	1.000 (1.000)
5. Work	−1.30 (3.48)	−0.19 (3.22)	0.48 (3.24)	4.13 (5.12)	0.029 (0.013)	0.26 (0.27)	0.023 (0.009)	0.636 (0.632)
6. Recreation	−0.83 (3.71)	0.63 (3.42)	0.36 (2.69)	0.63 (0.63)	0.544 (0.541)	0.05 (0.04)	1.000 (1.000)	1.000 (1.000)
7. Learning	−1.57 (2.98)	1.89 (2.21)	1.92 (2.36)	15.53 (16.23)	**<0**.**001 (<0**.**001)**	0.58 (0.54)	**<0**.**001 (<0**.**001)**	1.000 (1.000)
8. Creativity	1.17 (3.63)	2.56 (2.62)	2.48 (2.45)	3.148 (2.990)	0.062 (0.067)	0.22 (0.18)	0.189 (0.186)	1.000 (1.000)
9. Social service	0.07 (2.49)	1.11 (2.28)	1.28 (2.64)	2.864 (3.887)	0.077 (0.032)	0.20 (0.22)	0.184 (0.090)	1.000 (1.000)
10. Love relationships	−1.03 (4.06)	−0.33 (3.55)	−0.20 (3.20)	2.478 (1.837)	0.106 (0.178)	0.18 (0.12)	0.097 (0.185)	1.000 (1.000)
11. Friendships	1.63 (3.00)	2.52 (2.83)	2.28 (2.98)	1.067 (1.170)	0.360 (0.325)	0.09 (0.08)	0.657 (0.570)	1.000 (1.000)
12. Relationships with children	−0.17 (3.30)	−0.37 (3.51)	0.04 (3.55)	0.284 (0.287)	0.756 (0.753)	0.02 (0.02)	1.000 (1.000)	1.000 (1.000)
13. Relationships with relatives	0.80 (3.42)	2.00 (3.33)	2.36 (2.72)	3.367 (1.681)	0.052 (0.205)	0.23 (0.11)	0.042 (0.224)	1.000 (1.000)
14. Home	−1.53 (4.06)	−1.48 (3.85)	−1.88 (3.30)	0.412 (0.217)	0.667 (0.806)	0.04 (0.02)	1.000 (1.000)	1.000 (1.000)
15. Neighbourhood	−0.07 (3.36)	1.11 (3.06)	0.48 (2.65)	1.114 (1.112)	0.345 (0.343)	0.09 (0.07)	1.000 (1.000)	1.000 (1.000)
16. Community	−0.77 (2.78)	−1.04 (2.92)	−1.20 (2.74)	0.272 (0.275)	0.764 (0.762)	0.02 (0.02)	1.000 (1.000)	1.000 (1.000)

Results from the intent-to-treat (ITT) population using last observation carried forward (LOCF) are presented within parentheses, *n* = 30

Bold values indicate statistically significant *P* value

#### Associations between symptomatic and functional improvements

Pearson’s product moment correlation coefficients (*r*) for symptom and functional rating scales are depicted in Table 6. ADHD symptoms measured by both the inattention and the hyperactivity/impulsivity subscales of the investigator-rated CAARS: O-SV, as well as the self-rated ASRS, correlated negatively with global functioning measured by GAF. The associations were evident from study week 16 onwards, being the strongest at open-label endpoint after 52 weeks of treatment, with correlation coefficients (*r*) ranging from −0.483 to −0.736, as presented in Table 6. Further, both inattention subscales of CAARS: O-SV and ASRS associated stronger with GAF than the hyperactivity/impulsivity subscales. On the other hand, global symptom severity measured by CGI-S was negatively associated with GAF already from baseline (*r* = −0.486, *P* = 0.006), with increased convergence over time, to be most consistent by endpoint at week 52 (*r* = −0.885, *P* < 0.001). Finally, QOLI correlated weaker with symptomatic improvements than did GAF. The only significant association of QOLI and symptomatic improvement was the Goals and values domain, which correlated negatively with the inattention subscales of both CAARS: O-SV and ASRS. However, the associations were significant only by the open-label endpoint at week 52 (CAARS: O-SV-Inattention, *r* = −0.414, *P* = 0.040; ASRS-Inattention, *r* = −0.551, *P* = 0.004).

**Table 6:** Pearson’s product moment correlation coefficients (*r*) between ratings of symptom severity and functional outcomes over the cumulated 52-week study period

	GAF Baseline	QOLI Goals and values Baseline	QOLI Learning Baseline	GAF Week 16	QOLI Goals and values Week 16	QOLI Learning Week 16	GAF Week 52	QOLI Goals and values Week 52	QOLI Learning Week 52
*CAARS: O-SV, baseline, n* *=* *30*									
Inattention	−0.340 (0.066)	−0.288 (0.123)	−0.207 (0.272)						
Hyperactivity/impulsivity	−0.112 (0.555)	−0.071 (0.709)	0.212 (0.261)						
*CAARS: O-SV, week 16, n* *=* *27*									
Inattention				−0.747 (<0.001)**	0.318 (0.105)	−0.164 (0.414)			
Hyperactivity/impulsivity				−0.675 (<0.001)**	0.131 (0.515)	−0.104 (0.604)			
*CAARS: O-SV, week 52, n* *=* *25*									
Inattention							−0.736 (<0.001)**	−**0.414 (0**.040)*	−0.281 (0.174)
Hyperactivity/impulsivity							−0.483 (0.015)*	−0.074 (0.724)	0.078 (0.712)
*ASRS, baseline, n* *=* *30*									
Inattention	−0.306 (0.100)	−0.010 (0.959)	0.084 (0.659)						
Hyperactivity/impulsivity	−0.275 (0.142)	0.078 (0.682)	0.078 (0.683)						
*ASRS, week 16, n* *=* *27*									
Inattention				−0.550 (0.003)**	0.091 (0.652)	−0.136 (0.498)			
Hyperactivity/impulsivity				−0.503 (0.007)**	0.019 (0.925)	−0.178 (0.374)			
*ASRS, week 52, n* *=* *25*									
Inattention							−0.624 (0.001)**	−0.551 (0.004)**	−0.296 (0.150)
Hyperactivity/impulsivity							−0.577 (0.003)**	−0.229 (0.271)	−0.140 (0.506)
CGI-S, baseline, *n* = 30	−0.486 (0.006)**	−0.319 (0.086)	−0.015 (0.938)						
CGI-S, week 16, *n* = 27				−0.846 (<0.001)**	0.145 (0.469)	−0.333 (0.090)			
CGI-S, week 52, *n* = 25							−0.885 (<0.001)**	−0.320 (0.118)	−0.319 (0.120)

Bold values indicate the significant correlation coefficients and their corresponding *P* values

*P* values are reported within parentheses

* *P* < 0.05; ** *P* < 0.01

#### Associations between investigators’ and self-ratings of ADHD symptoms

Investigator-rated ADHD symptoms by the total sum-score of CAARS: O-SV correlated strongly with self-reported ADHD symptoms by the total sum-score of ASRS, at all assessments (T1, T2 and T3). The Pearson’s product moment correlation coefficients (*r*) increased over time, from baseline (T1) until open-label endpoint at week 52 (T3), ranging from 0.473 to 0.730 (all *Ps* < 0.01) as shown in Table 7.

**Table 7:** Pearson’s product moment correlation coefficients (*r*) between investigator-ratings of ADHD symptoms by CAARS: O-SV and self-ratings of ADHD symptoms by ASRS and changes in symptom frequencies over the cumulated 52-week study period

CAARS: O-SV, sum-score	ASRS, sum-score baseline	ASRS, change in sum-score from baseline to week 16	ASRS, sum-score week 16	ASRS, change in sum-score from baseline to week 52	ASRS, sum-score week 52
Baseline, *n* = 30	0.473 (0.008)**				
Changes from baseline to week 16		0.545 (0.003)**			
Week 16, *n* = 27			0.599 (0.001)**		
Changes from baseline to week 52				0.697 (<0.001)**	
Week 52, *n* = 25					0.730 (<0.001)**

*P* values are reported within parentheses

** *P* < 0.01

## Discussion

Recently, we reported primary findings from the first controlled trial that evaluated treatment with OROS-methylphenidate in prison inmates with ADHD and coexisting disorders [40]. This trial was carried out in 30 adult males who served conviction mainly due to violent or drug-related offences, and therefore were hosted at a high-security prison. An initial 5-week randomized, double-blind, placebo-controlled phase with OROS-methylphenidate delivered 72 mg daily was followed by an 47-week open-label extension with OROS-methylphenidate delivered at a flexible daily dosage of up to 1.3 mg/kg body weight in all participants. During the initial phase, OROS-methylphenidate outperformed placebo robustly (*P* < 0.00 l, Cohen’s *d* = 2.17) in improvements in ADHD symptoms, global severity and global functioning. These outcomes continued to improve significantly within participants during the open-label extension.

In the present study, we evaluated the long-term effectiveness and maintenance of improvements from OROS-methylphenidate over the cumulated 52 weeks of treatment. Both verbal and visuospatial working memory, and abstract verbal reasoning improved significantly within participants, as well as cognition-related measures by CPTs, motor activity and a few domains of self-reported quality of life. Improvements mainly occurred between baseline and study week 16, with maintenance or further improvements in outcomes until open-label endpoint at week 52. A vast majority of participants took part in accredited treatment programmes as well as educational activities, and no substance misuse was detected during the course of the study. The post hoc correlation analyses suggested that improvements in ADHD symptoms and global symptom severity were all strongly associated with functional improvement measured by GAF. Quality of life measured by QOLI was on the other hand lesser associated with symptomatic improvement than GAF. However, the QOLI domain of Goals and values was related to improvements in attention, but only significantly at open-label endpoint. Finally, investigators’ and self-ratings of ADHD symptoms were significantly associated from baseline onwards, being most convergent by open-label endpoint at week 52.

Previous studies have shown deficits in temporal, parietal and frontal lobe function in both children and adults with ADHD [2, 55]. Go/no go tasks used in CPTs have demonstrated significant differences in commission errors, variability of reaction time, omission errors and perceptual sensitivity between individuals with ADHD and controls. These deficits were associated with fMRI findings of hypoactivation in specific brain regions in those with ADHD, alongside hyperactivation in other areas, suggesting the hyperactive regions to compensate for executive dysfunction [10]. Recent fMRI studies have also discovered an intrinsic organizational system of brain activity, with a proposed functional connectivity between brain regions in several temporally anti-correlated networks [9]. Temporal anticorrelation means that the so-called default mode network is active during the resting state, as opposed to the ‘task mode network’, which is active during task performance. Consequently, both networks are not supposed to be active at the same time.

One of the most consistent findings among individuals with ADHD is increased reaction time variability as measured by CPTs. This reaction time variability is proposed to reflect infrequent lapses of attention, related to insufficient suppression of the default mode network [9]. That means the default mode network seems to be active and interfering with the task mode network during task performance. This is in line with a recent study, suggesting that individuals with ADHD have a relative weakness in suppressing activity in the default mode network during performance of a working memory task [56]. Our findings of methylphenidate improving sustained attention and reaction time variability, while also improving ADHD symptoms, lend support to these previous findings. Our observations are also consistent with a recent proposal of stimulants facilitating the deactivation of the default mode network, with corresponding decreases in lapses of attention, thus ameliorating symptoms of inattention [2].

In the present study, both verbal and visuospatial working memory improved over time. This is in consistence with a study by Fallu that reported improvements in working memory functions within adults with ADHD that participated in an open-label trial evaluating OROS-methylphenidate [37].

Further, in the present study, objective quantification of motor activity by QbTest showed a considerable motor hyperactivity within participants. At baseline, they differed by as much as 2–3 standard deviations as compared to the norm group. The presence of objectively measured motor hyperactivity in adults with ADHD is consistent with a previous report by Lis et al. [4]. Both these observations challenge the commonly held view of motor hyperactivity not being of a concern in adults. The reason for the substantially increased motor activity observed in the present study is not obvious. It might be that prison inmates with ADHD represent a specific, homogeneous group of ADHD, with substantially persistent and pervasive symptoms and impairments across modalities. This suggestion is, at least in part, supported by our previous report of prison inmates being more symptomatic and dysfunctional as compared to a group of adults with ADHD from a psychiatric outpatient clinic [15]. However, more research is warranted to clarify this issue. Moreover, OROS-methylphenidate significantly decreased motor activity over time, although not to the extent that motor activity was normalized as compared to the norm. These findings are in line with a placebo-controlled study conducted in children with ADHD that observed atomoxetine to significantly decrease motor hyperactivity as compared to placebo [57]. Our findings are also in consistence with a study by Vogt and Williams [58]. They reported a robust treatment response on motor activity measured by QbTest, in a group of children and adolescents with hyperkinetic disorder who were administered a single dose of methylphenidate.

The Swedish Prison and Probation Service is part of the judicial system. Their primary aims are both to reduce recidivism in offences and to increase safety in society. In order to reduce recidivism, they provide various accredited treatment programmes, mainly addressing offending in general, violence and addiction. To increase the chances for inmates of obtaining a job after conditional release, they are provided work, vocational training and educational programmes. The educational programmes aim at increasing basic skills such as reading, writing and mathematics, preferably at the primary school level. The participants of the present study were at baseline substantially symptomatic from ADHD and coexisting disorders, including lifetime substance use disorder. They were also psychosocially dysfunctional and presented a very low educational level. A vast majority (83 %) had fulfilled 9 years of compulsory school or less, and 80 % had received educational support during childhood. Encouraging, as many as 87 % of participants took part in educational programmes, and 80 % took part in at least one accredited treatment programme. Long-term follow-ups will be performed to explore if taking part in the present study will be followed by reduced recidivism in criminality and substance abuse among participants.

Once imprisoned, individuals with untreated ADHD constitute a challenge by their aggressive behaviour [17, 59, 60]. Correctional officers find these inmates difficult to manage, both at the prison wings and in treatment programmes. Aggressive behavioural disturbances will lead to reports on critical incidents, often followed by formal sanctions, meaning that inmates with ADHD will be less likely considered for early release. In a previous study [59], inmates with ADHD were accounted for eight times more reports on critical incidents than inmates without ADHD. When controlled for antisocial personality disorder, inmates with ADHD still accounted for six times more reports than other inmates. The increased risk for aggressive behaviour was found to be related to factors such as persistence of ADHD symptoms, impulsivity, mood instability, low frustration tolerance and a disorganized/chaotic personality style. Therefore, effective treatment combinations are warranted for prison inmates with ADHD in order to reduce symptoms, improve control of behaviour and affect regulation, as well as to improve prosocial skills [60].

Considering the challenges and costs from handling aggressive inmates, it is also of importance to evaluate whether treatment with stimulants influences aggressive institutional behaviour. In the present study, we observed that critical incidents decreased during the study compared with the year before. However, because of methodological considerations, we did not employ inferential statistics. Therefore, this issue still needs to be explored in future studies. However, the vast majority of participants took part in treatment programmes and educational activities. For many of them, this was the first time they succeeded to attend programmes. Our results are indeed promising and suggest that stimulant treatment could be a useful part of a more comprehensive intervention approach. To successfully benefit from psychological treatment, we consider it essential to be able to concentrate, remain seated, and process and remember the information presented at the session. Pharmacological treatment could therefore facilitate for inmates to take part in psychological interventions addressing ADHD and prosocial competence, such as R&R2 for ADHD Youths and Adults [61].

Individuals with ADHD often self-report impairments in quality of life [62]. Therefore, it is important to evaluate the potential for stimulants to improve aspects of quality of life. In the present study, we used QOLI, a general self-reported questionnaire considered to apply to both non-psychiatric and psychiatric populations. Most previous studies that assessed QOLI, used a weighted, global index of subjective life satisfaction, derived from the 16 specific domains of QOLI. However, we decided to evaluate changes in the specific domains instead of using the single global index, since life satisfaction may differ between specific domains, and several domains were considered non-relevant for prison inmates and difficult to change within the restricted prison environment. We observed significant improvements in the domains of Goals and values, and Learning, respectively. Also, domains of health, self-regard, work, and relationships with relatives improved over time, although not significantly. Importantly, participation in the educational programmes seems to have improved self-reported quality of life, since the domain of Learning was the one to improve the most. Also, the domain of Goals and values in life improved substantially over time, and at open-label endpoint, it was significantly associated with improvements in attention subscales. How can we interpret these findings? We suggest that symptomatic and functional improvements, together with new experiences of succeeding at school and in treatment programmes, as well as being able to control behaviour instead of being reported for critical incidents, contributed to increased self-respect and an improved sense of internal locus of control. If a life situation becomes possible to change, this could raise hope for the future. Goals in life that previously seemed impossible to reach might become meaningful to consider. Also, the observed improvements in cognition, such as working memory and abstract verbal reasoning, might have facilitated in this change of view.

Improvements in self-reported quality of life as observed in this study are consistent with previous reports of improved quality of life by treatment, although previous studies used other quality of life questionnaires [62].

As most previous trials have demonstrated the effectiveness of stimulants on ADHD symptoms, it was not fully understood if symptomatic improvements translated into functional improvements. However, the relationships between symptomatic and functional improvements were recently addressed in a few studies, suggesting a relationship [27, 36–38]. Our post hoc analysis implied a strong association between improvements in ADHD symptoms, rated by both investigators and participants and functional improvements by the investigator-rated GAF, which is in favour of the previous suggested relationships between symptoms and functions. However, quality of life domains of QOLI were weaker associated with symptomatic improvement than GAF. In fact, Goals and values was the only domain that significantly related to symptomatic improvement, and only at the open-label endpoint. This weaker association between quality of life and symptomatic improvements might be related to the use of QOLI, a general questionnaire rather than ADHD specific. It might also be that QOLI was insufficiently sensitive to detect changes within participants with ADHD specifically. As suggested previously, other explanations could be that QOLI comprises domains, either non-relevant and/or difficult to influence within a prison setting.

The post hoc analysis suggested a strong association between investigators’ and participant ratings of ADHD symptoms, which is in line with a previous report by Adler et al. [39], who examined the psychometric properties of the CAARS scale used in two studies of adult ADHD patients that were randomized to 10-week treatment with atomoxetine or placebo. The authors found that investigators’ and participant ratings of ADHD symptoms (CAARS scales) were highly variable at baseline, but the interrater reliability increased substantially by the endpoint of treatment. This was suggested to be reflective of the decreased frequency of ADHD symptoms from effective treatment, thereby reducing the variability in symptom reports. It was also suggested that previously untreated participants increased the ability over time to assess and report their ADHD symptoms in a manner similar to the investigators. Notably, in the present study, the variability also seemed to be substantially reduced in cognition-related measures, paralleling the reduced variability in symptom reports (Tables 3, 4; Fig. 3).

Some limitations of the present study need to be considered when interpreting the results. The 47-week open-label extension lacked a comparator, as only the initial 5-week period was placebo-controlled. As a consequence, you would expect larger effects from uncontrolled conditions, not adjusting for non-specific effects as compared to a placebo-controlled trial. On the other hand, results of the neuropsychological tests were all compared to norm group data, which could be viewed as an indirect comparator. Further, the study sample was small, thus limiting the range of statistical analyses being performed. Also, the study population comprised prison inmates with ADHD and coexisting disorders, including personality disorders, lifetime substance use disorder, autism-spectrum disorder, antisocial behaviour, anxiety and affective disorders. Results may therefore not be generalizable to other adults with ADHD but without the same spectrum of coexisting disorders. As this trial is the first of its kind, conducted within a prison environment, we are not able to compare our results with other similar studies. However, when we compare the results of the present study with results from previous studies conducted in adults with ADHD, preferably from the general psychiatry, the effect size of the present study (*d* = 2.17) by far exceeds the effect sizes reported by previous studies. Most of these studies did, however, exclude participants with substantial coexisting disorders, thus not reflecting ADHD in the general population. Therefore, based on our findings, we suggest that adults with ADHD and coexisting disorders might improve more from treatment than adults without coexisting disorders. As the trial was conducted within a prison, treatment was strictly controlled, as was compliance, thereby probably contributing to the large effect sizes as seen in this study. These results could therefore be difficult to translate into regular clinical practice without the same controlled conditions, thus likely resulting in a lower compliance to treatment. Moreover, there were only single baseline assessments of neuropsychological tests, which imply a risk for effects of repeated testing. However, the effect sizes indicated very large improvements by test norm standards that most likely could not fully be explained by effects of repeated testing alone. Also, effects were observed on tests in which effects of repeated testing were not expected, such as abstract verbal reasoning (Similarities), and verbal and non-verbal working memory (Digit Span and Span Board, respectively). Another limitation was that we used Similarities as a measure of specificity. At forehand, we did not expect changes in Similarities by OROS-methylphenidate treatment. However, the ability of abstract verbal reasoning as measured by Similarities, improved significantly over time, thus limiting the usefulness of Similarities as a specificity measure. On the other hand, it was encouraging that abstract verbal reasoning actually improved, eventhough it was unexpected.

On the other hand, this study also has strengths. This was the first study to evaluate OROS-methylphenidate as treatment for prison inmates with ADHD and coexisting disorders, and it is so far one of few long-term studies in adults with ADHD that observed a robust treatment response, both in the short term and in the long term. Inclusion criteria were broader, allowing for the presence of coexisting disorders, thus increasing generalizability of results. The flexible dosing during the open-label extension aimed at reflecting regular clinical practice. Since ADHD is a complex and heterogeneous disorder, we aimed at exploring outcomes from a broader perspective, incorporating several aspects of improvement, such as symptoms, global functioning, cognition, motor activity, institutional behaviour and quality of life. We also conducted post hoc analyses that evaluated the translation of symptomatic improvements into functional improvements, and the associations between investigators’ and self-ratings of ADHD symptoms. Based on our findings of improved cognition in participants, we suggest a broadening of outcome measures in future clinical trials to also include objective measurements such as CPTs, with tracking of motor activity. Moreover, the high correlations between investigators’ and self-reported ADHD symptoms, as well as between symptom ratings and functional ratings, imply self-reported ADHD symptom scales to be reliable. An increased use of self-reported symptom scales, preferably combined with, for instance, the more easily observer-rated CGI, might facilitate monitoring of pharmacological treatment in regular clinical practice and might be cost-saving as well. Further, as the results on the Conners’ CPT (see Fig. 3) almost normalized as compared to the norm by treatment with OROS-methylphenidate, it might be that more ecologically valid outcome measures need to be used in future trials when evaluating ‘add-on’ treatments to pharmacological treatment in a multimodal approach, to reduce the possibility of ceiling effects.

In conclusion, OROS-methylphenidate was an effective and overall safe treatment for adult male prison inmates with ADHD and coexisting disorders, both in the short term and in the long term. As this was the first study evaluating stimulant treatment for prison inmates with ADHD within a prison environment, our results need to be confirmed.
